# Demonstration of the impact of COVID-19 on metabolic associated fatty liver disease by bioinformatics and system biology approach

**DOI:** 10.1097/MD.0000000000034570

**Published:** 2023-09-01

**Authors:** Tengda Huang, Dawei Zheng, Yujia Song, Hongyuan Pan, Guoteng Qiu, Yuchu Xiang, Zichen Wang, Fang Wang

**Affiliations:** a Innovation Center of Nursing Research, Nursing Key Laboratory of Sichuan Province, West China Hospital, Sichuan University, Sichuan, Chengdu, China; b Division of Liver Surgery, Department of General Surgery and Laboratory of Liver Surgery, and State Key Laboratory of Biotherapy and Collaborative Innovation Center of Biotherapy, West China Hospital, Sichuan University, Chengdu, China; c The College of Life Sciences, Sichuan University, Chengdu, China; d State Key Laboratory of Biotherapy, Sichuan University, Chengdu, China.

**Keywords:** COVID-19, differentially expressed genes, drug molecules, MAFLD, protein-protein interaction

## Abstract

**Background:**

Severe coronavirus disease 2019 (COVID-19) has caused a great threat to human health. Metabolic associated fatty liver disease (MAFLD) is a liver disease with a high prevalence rate. Previous studies indicated that MAFLD led to increased mortality and severe case rates of COVID-19 patients, but its mechanism remains unclear.

**Methods:**

This study analyzed the transcriptional profiles of COVID-19 and MAFLD patients and their respective healthy controls from the perspectives of bioinformatics and systems biology to explore the underlying molecular mechanisms between the 2 diseases. Specifically, gene expression profiles of COVID-19 and MAFLD patients were acquired from the gene expression omnibus datasets and screened shared differentially expressed genes (DEGs). Gene ontology and pathway function enrichment analysis were performed for common DEGs to reveal the regulatory relationship between the 2 diseases. Besides, the hub genes were extracted by constructing a protein-protein interaction network of shared DEGs. Based on these hub genes, we conducted regulatory network analysis of microRNA/transcription factors–genes and gene - disease relationship and predicted potential drugs for the treatment of COVID-19 and MAFLD.

**Results:**

A total of 3734 and 589 DEGs were screened from the transcriptome data of MAFLD (GSE183229) and COVID-19 (GSE196822), respectively, and 80 common DEGs were identified between COVID-19 and MAFLD. Functional enrichment analysis revealed that the shared DEGs were involved in inflammatory reaction, immune response and metabolic regulation. In addition, 10 hub genes including SERPINE1, IL1RN, THBS1, TNFAIP6, GADD45B, TNFRSF12A, PLA2G7, PTGES, PTX3 and GADD45G were identified. From the interaction network analysis, 41 transcription factors and 151 micro-RNAs were found to be the regulatory signals. Some mental, Inflammatory, liver diseases were found to be most related with the hub genes. Importantly, parthenolide, luteolin, apigenin and MS-275 have shown possibility as therapeutic agents against COVID-19 and MAFLD.

**Conclusion:**

This study reveals the potential common pathogenesis between MAFLD and COVID-19, providing novel clues for future research and treatment of MAFLD and severe acute respiratory syndrome coronavirus 2 infection.

## 1. Introduction

Induced by severe acute respiratory syndrome coronavirus 2 (SARS-CoV-2), coronavirus disease 2019 (COVID-19) has become a great threat to public health.^[1]^ Since the end of 2019, there have been more than 62 million confirmed cases of COVID-19 and more than 6.58 million deaths in a total of 213 countries as of December 2022, which has exerted an enormous impact on the medical industry and whole society.^[2]^ SARS-CoV-2 is constructed by nucleocapsid (N protein), envelope (E protein), membrane (M protein), and spike (S protein). The N protein combined with the viral genomic RNA is packed in the virus. Structural proteins S, E, and M construct the membrane of the virus.^[3]^ By binding the S protein to a receptor called angiotensin converting enzyme 2 (ACE2), the virus enters the targeted cell in the primary step.^[4]^ ACE2s are primarily found on the surface of ciliated bronchial cells, lung alveolar epithelial cells, and endothelial cells.^[5]^ Thus, the most common clinical symptoms of COVID-19 are dry cough, dyspnea, and pneumonia. Furthermore, ACE2 is also found in the membrane of cells in the liver,^[6]^ intestines, kidney, testis, gallbladder, and heart,^[5]^ which accounts for why COVID-19 is a systemic disease and can cause shock, acute cardiac injury, multiple organ failure and eventual death.^[7]^ Therefore, it is of urgency to study the mechanism of how COVID-19 interacts with other diseases.

The liver is one of the crucial metabolic organs in the body due to its biological functions in lipid metabolism.^[8]^ Caused by toxic lipid species accumulating in liver, inducing hepatocellular stress, injury and death, and leading to fibrogenesis and genomic instability, metabolic associated fatty liver disease (MAFLD) is a disease whose prevalence rate among adults is 25% worldwide.^[9,10]^ The occurrence of MAFLD is related to obesity, cardiovascular disease,^[11]^ and the differential expression of genes involved in lipid droplet biology.^[12]^ With histological abnormalities like bland steatosis, steatohepatitis, hepato-fibrosis, and cirrhosis, MAFLD is at risk for further progression to nonalcoholic steatohepatitis (NASH), liver fibrosis, cirrhosis, and liver cancer.^[13]^ Research conducted by Bing Guo et al demonstrated that exposure to environmental air pollution over a long period might increase the probability of MAFLD, which indicates that MAFLD may correlate with respiratory diseases.^[14]^ A spectrum of studies have revealed the existence of a relationship between COVID-19 and MAFLD, including a synergistic effect of MAFLD for severe COVID-19 rate in the patient, an increased risk of disease progression among patients with COVID-19 caused by MAFLD and exceptionally high mortality rates from COVID-19 among patients with alcohol-related liver disease.^[14–17]^

Bioinformatics is a discipline concerning acquiring, storing, visualizing, and interpreting medical or biological data and coping with genetic information with research, development, or application of computational tools and approaches.^[18]^ Systems biology is an approach in biomedical research to understanding the interaction of the organism, tissue, or cell in a systematic and integral view.^[19]^ Bioinformatics and system biology approach open a new way tounderstand biological problems.

**Figure 1.**
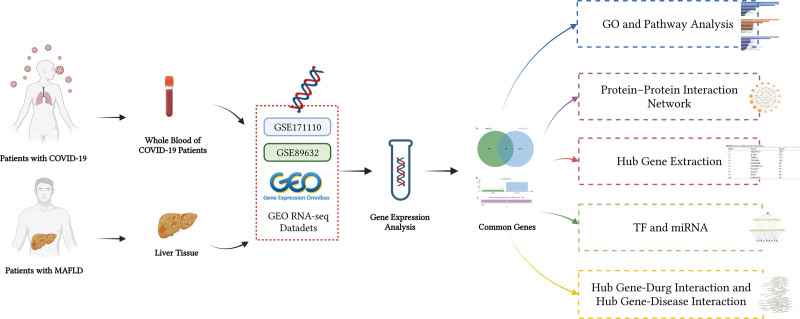
Schematic diagram of the overall workflow of the study.

Due to the high prevalence rate of MAFLD and the worldwide epidemic of COVID-19, it is an urgent clinical issue to explore the impact of COVID-19 on MAFLD and find potential drug molecules. Herein, the research attempts to investigate the potential genetical association and screened out the possible drug molecules between MAFLD and COVID-19 by bioinformatics and system biology approach. This study may provide new insights into common pathogenesis of the 2 diseases. The flowchart (Fig. 1) clarifies the rationale of the study.

## 2. Materials and methods

### 2.1. Acquirement of datasets

To reveal the genetic correlation between COVID-19 and MAFLD, this research selected 2 datasets from gene expression omnibus datasets (GEO) (https://www.ncbi.nlm.nih.gov/geo/) of National Center for Biotechnology Information (https://www.ncbi.nlm.nih.gov/). The COVID-19 accession id is GSE196822,^[20]^ including mRNA isolated from whole blood of 34 COVID-19 patients and 9 healthy controls. Similarly, the MAFLD accession id is GSE183229,^[21]^ which contained transcriptome profiling from liver tissues of 9 normal controls and 7 MAFLD patients. The datasets abovementioned were sequenced by GPL20301 Illumina HiSeq 4000 (Homo sapiens).

### 2.2. Filtration of differentially expressed genes (DEGs) among COVID-19 and MAFLD

DEG refers to the gene whose expression level is statistically different from the gene from another group. We conducted a differential analysis to screen out the DEGs among COVID-19 and MAFLD. Genes with |log_2_ Fold Change|≥1 and false discover rate <0.05 were selected as DEGs from GSE196822 and GSE183229 datasets with DEseq2 package^[22]^ in R software, respectively. Herein, the overlapped DEG means the DEG both appear in COVID-19 and MAFLD. An online Venn analysis tool named Jvenn^[23]^ was applied to acquire overlapped DEGs of GSE196822 and GSE183229 datasets.

### 2.3. Annotation of the biological function and signaling pathways of overlapped DEGs

Enrichment analysis^[24]^ is an analytical endeavor that enriches biological knowledge, including signaling pathways, biological mechanisms, etc. Constructed by 3 ontologies molecular function (MF), cellular component (CC) and biological process, gene ontology (GO) is a classification system of gene function aiming at describing the properties of the genes and their production.^[25]^ WikiPathways is a comprehensive database concerning biological knowledge in pathways,^[26]^ Kyoto Encyclopedia of Genes and Genomes (KEGG), a genomic database, provides the knowledge of gene functions,^[27]^ and Reactome is a database consisting of the data and models about cellular processes.^[28]^ All these databases abovementioned can promote the understanding of the biological connection and function of these genes.^[27]^ To investigate the potential functions and pathways in these overlapped DEGs, we conducted an enrichment analysis on them and uncovered their biological mechanisms and signaling pathways of them, utilizing the clusterProfiler package in the EnrichR online platform (http://amp.pharm.mssm.edu/Enrichr).

### 2.4. Protein-protein interaction (PPI) network

Proteins are the most crucial agents of biological functions in organisms, and they do not perform their functions respectively but rather interact with other proteins and molecules.^[29]^ A comprehensive understanding of PPI can aid in depicting the association of overlapped DEGs. Utilizing STRING,^[30]^ we uploaded overlapped DEGs to the program for the assessment of critical physical and functional associations of PPI and constructing the PPI network of frequent DEGs with a composite score larger than 0.4. The visualization of the investigation was realized using Cytoscape.^[31]^

### 2.5. Extraction of hub genes

Based on the PPI network, we utilized CytoHubba (https://apps.cytoscape.org/apps/cytohubba), the plugin of Cytoscape for ranking and extracting crucial, potential or targeted components of a biological network,^[32]^ to select the hub genes. Hereafter, the degree algorithm, a widely used centrality criteria, was put into practice to filtrate the first few genes. Then we used the CytoHubba software to rank the shortest accessible path among the hub genes.

### 2.6. The identification of hub genes related transcription factor (TF) and micro-RNA (miRNA)

TF is a protein able to determine the process and rate of DNA transcription by binding to the specific sequence of DNA.^[33]^ MiRNA is a small and single-stranded RNA molecule capable of pairing up with the complementary mRNA, leading to their cleavage and destabilization and silencing the gene.^[34]^ Either of these molecules plays an important role in adjusting gene expression. JASPAR (http://jaspar.genereg.net) is a database storing manually curated TF binding profiles as position frequency matrices.^[35]^ Position frequency matrices summarize occurrences of each nucleotide at each position in a set of observed TF-DNA interactions and are capable of being put into practice to scan any DNA sequence to predict TF binding sites.^[36]^ Via the tool of NetworkAnalyst,^[37]^ we selected the topologically feasible TFs from the JASPAR database and bound them with the hub genes. MiRTarBase is a well-known miRNA-target interaction comprehensive database.^[38]^ Using the tool of NetworkAnalyst, we recognized the miRNA interacting with hub genes from MiRTarBase.

### 2.7. Assessment of candidate drugs

Drug signatures database (DSigDB) is a genomic resource associated with drugs and compounds and their target genes for gene-set enrichment analysis.^[39]^ Via Enrichr and DSigDB, an analysis was conducted to medicine molecules based on the hub genes of COVID-19 and MAFLD to screen potential drug candidates with therapeutic value for MAFLD patients infected with SARS-CoV-2.

### 2.8. Analysis of gene-disease correlation

DisGeNET (http://www.disgenet.org/) is an integrated discovery platform aiming at coping with questions about the genetic underpinning of human diseases.^[40]^ DisGeNET and NetworkAnalyst were put into practice for a better understanding of diseases correlating to the hub genes.

## 3. Results

### 3.1. Recognition of DEGs and overlapped DEGs among MAFLD and COVID-19 datasets

**Figure 2.**
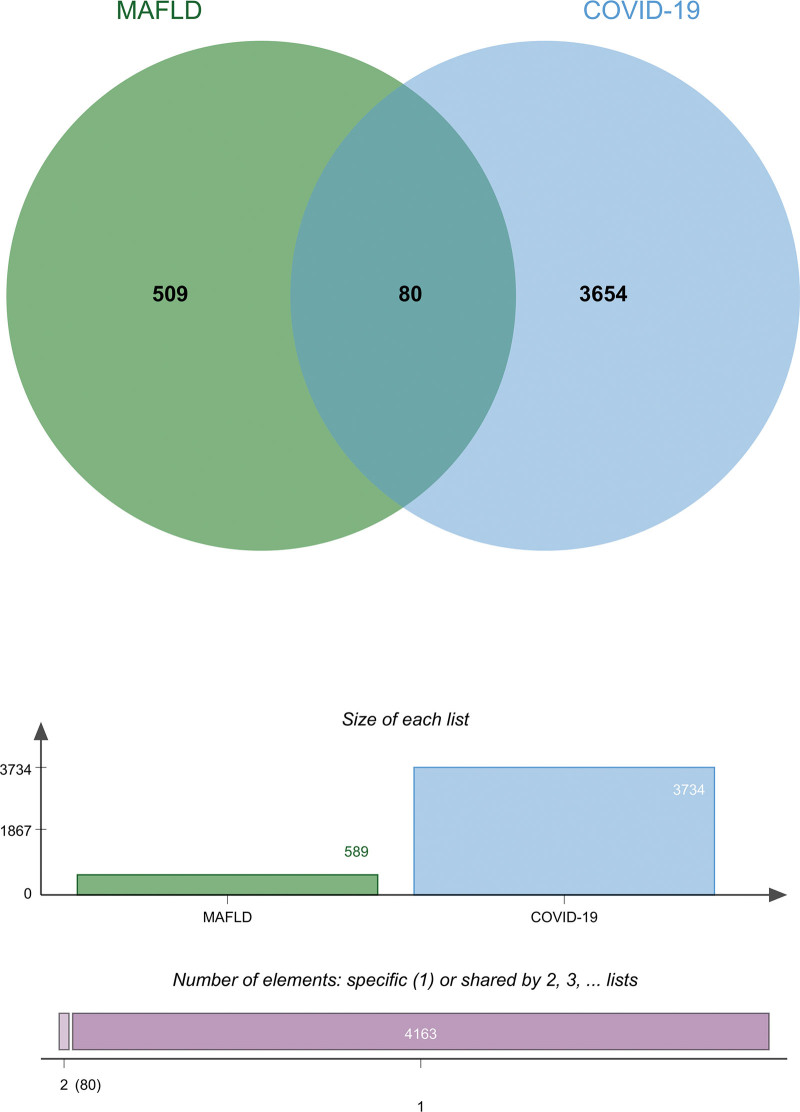
The Venn diagram shows the overlapped differentially expressed genes (DEGs) among coronavirus disease 2019 (COVID-19) and metabolic associated fatty liver disease (MAFLD).

Aiming to uncover the DEGs and overlapped DESs, we initially analyzed the COVID-19 datasets with the DEseq2 package and identified 3734 DEGs, including 1310 up-regulated DEGs and 2424 down-regulated DEGs. In the same way, 589 DEGs are revealed in the MAFLD datasets, with 252 up-regulated DEGs and 338 down-regulated DEGs included. All the basic information of the datasets of COVID-19 and MAFLD is listed in Table 1 summarily. Subsequently, Jvenn is utilized to acquire the overlapped DEGs among the aforementioned datasets, and 80 overlapped DEGs among MAFLD and COVID-19 were found in the Venn plot in Figure 2. These results suggest that there is a transcriptional regulatory correlation between MAFLD and COVID-19.

**Table 1:** Overview of the datasets about their details and DEGs amount.

Disease name	GEO accession	GEO platform	Total DEGs	Up-regulated DEGs	Down regulated DEGs
COVID-19	GSE196822	GPL20301	3734	1310	2424
MAFLD	GSE183229	GPL20301	589	252	338

COVID-19 = coronavirus disease 2019, DEGs = differentially expressed genes, GEO = gene expression omnibus datasets, MAFLD = metabolic associated fatty liver disease.

### 3.2. GO and pathway enrichment analysis

**Figure 3.**
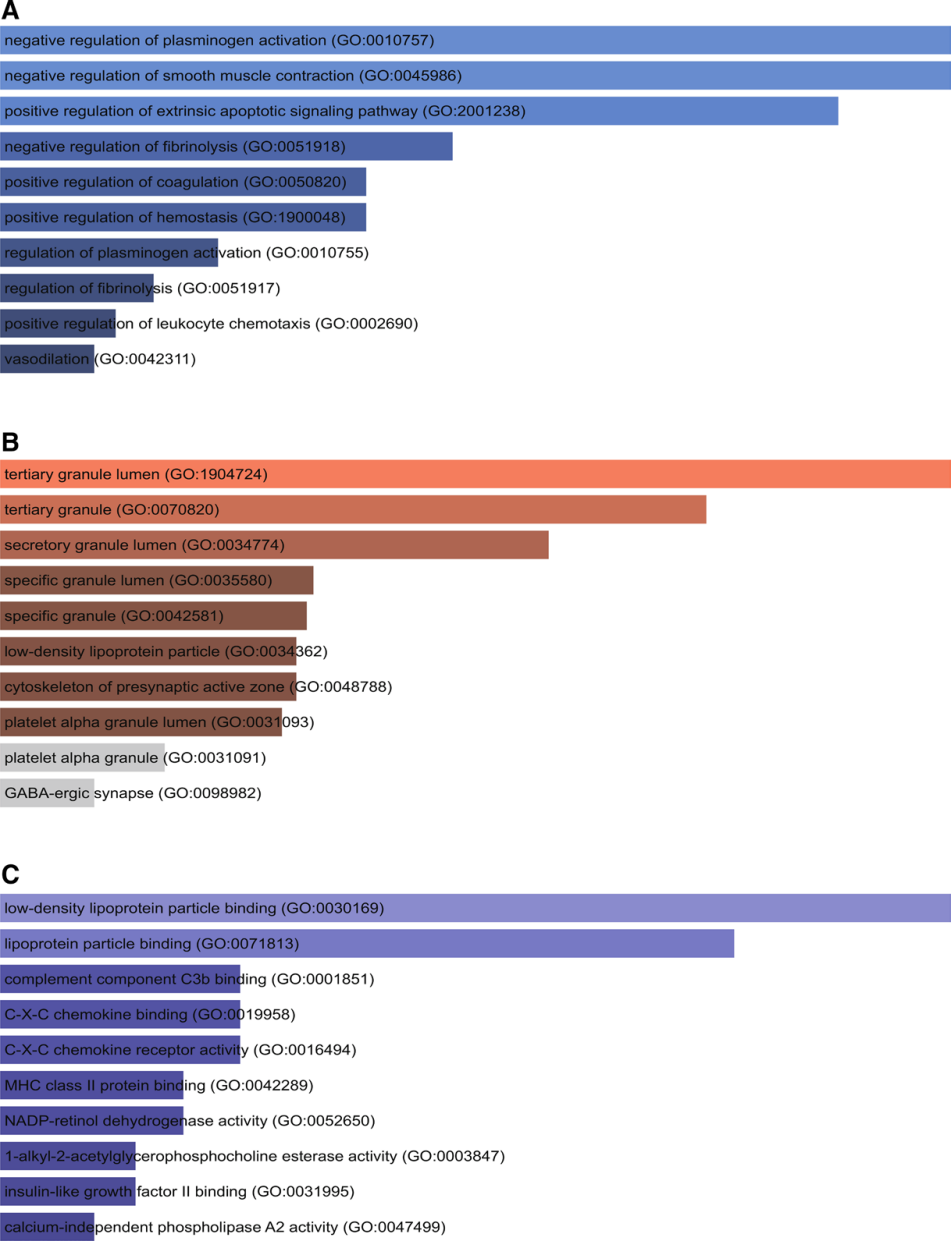
The horizontal bar charts depicting gene ontology (GO) analysis result of overlapped differentially expressed genes (DEGs) among coronavirus disease 2019 (COVID-19) and metabolic associated fatty liver disease (MAFLD): (A) biological progress; (B) cellular component; (C) molecular function.

To investigate the potential functions and pathways, we performed an enrichment analysis of overlapped DEGs. The top ten terms (CC, biological process, and MF) in GO analysis are listed in Table 2. Figure 3 depicts the GO analysis in a bar chart. The analysis reveals that biological functions regulated most significantly in each ontology respectively are negative regulation of plasminogen activation in biological progress, secretory granule lumen in CC, and RAGE receptor binding in MF.

**Table 2:** Top 10 terms and their details in each gene ontology (GO) enrichment analysis of overlapped differentially expressed genes (DEGs) among coronavirus disease 2019 (COVID-19) and metabolic associated fatty liver disease (MAFLD).

Category	Term	*P* value	Genes
Biological progress	Negative regulation of plasminogen activation (GO:0010757)	1.57E-04	SERPINE1/THBS1
	Negative regulation of smooth muscle contraction (GO:0045986)	1.57E-04	KCNMA1/ADRB2
	Positive regulation of extrinsic apoptotic signaling pathway (GO:2001238)	2.09E-04	TNFRSF12A/G0S2/THBS1
	Negative regulation of fibrinolysis (GO:0051918)	5.59E-04	SERPINE1/THBS1
	Positive regulation of coagulation (GO:0050820)	6.96E-04	SERPINE1/THBS1
	positive regulation of hemostasis (GO:1900048)	6.96E-04	SERPINE1/THBS1
	Regulation of plasminogen activation (GO:0010755)	.001016061	SERPINE1/THBS1
	Regulation of fibrinolysis (GO:0051917)	.001197688	SERPINE1/THBS1
	Positive regulation of leukocyte chemotaxis (GO:0002690)	.001319748	SERPINE1/PLA2G7/THBS1
	Vasodilation (GO:0042311)	.001393682	KCNMA1/ADRB2
Cellular component	Tertiary granule lumen (GO:1904724)	.001391889	TNFAIP6/CRISP3/PTX3
	Tertiary granule (GO:0070820)	.004250089	TNFAIP6/CRISP3/PTX3/GPR84
	Secretory granule lumen (GO:0034774)	.008737035	CRISP3/SERPINE1/PTX3/S100P/THBS1
	Specific granule lumen (GO:0035580)	.025592262	CRISP3/PTX3
	Specific granule (GO:0042581)	.026369178	CRISP3/PTX3/GPR84
	Low-density lipoprotein particle (GO:0034362)	.027670153	PLA2G7
	Cytoskeleton of presynaptic active zone (GO:0048788)	.027670153	PCLO
	Platelet alpha granule lumen (GO:0031093)	.029543092	SERPINE1/THBS1
	Platelet alpha granule (GO:0031091)	.050472005	SERPINE1/THBS1
	GABA-ergic synapse (GO:0098982)	.069631774	PCLO
Molecular function	Low-density lipoprotein particle binding (GO:0030169)	2.07E-03	STAB2/THBS1
	Lipoprotein particle binding (GO:0071813)	4.12E-03	STAB2/THBS1
	complement component C3b binding (GO:0001851)	1.98E-02	VSIG4
	C-X-C chemokine binding (GO:0019958)	1.98E-02	ACKR3
	C-X-C chemokine receptor activity (GO:0016494)	1.98E-02	ACKR3
	MHC class II protein binding (GO:0042289)	2.38E-02	FCRL6
	NADP-retinol dehydrogenase activity (GO:0052650)	2.38E-02	DHRS13
	1-alkyl-2-acetylglycerophosphocholine esterase activity (GO:0003847)	2.77E-02	PLA2G7
	Insulin-like growth factor II binding (GO:0031995)	2.77E-02	IGFBP2
	Calcium-independent phospholipase A2 activity (GO:0047499)	.031560821	PLA2G7

**Figure 4.**
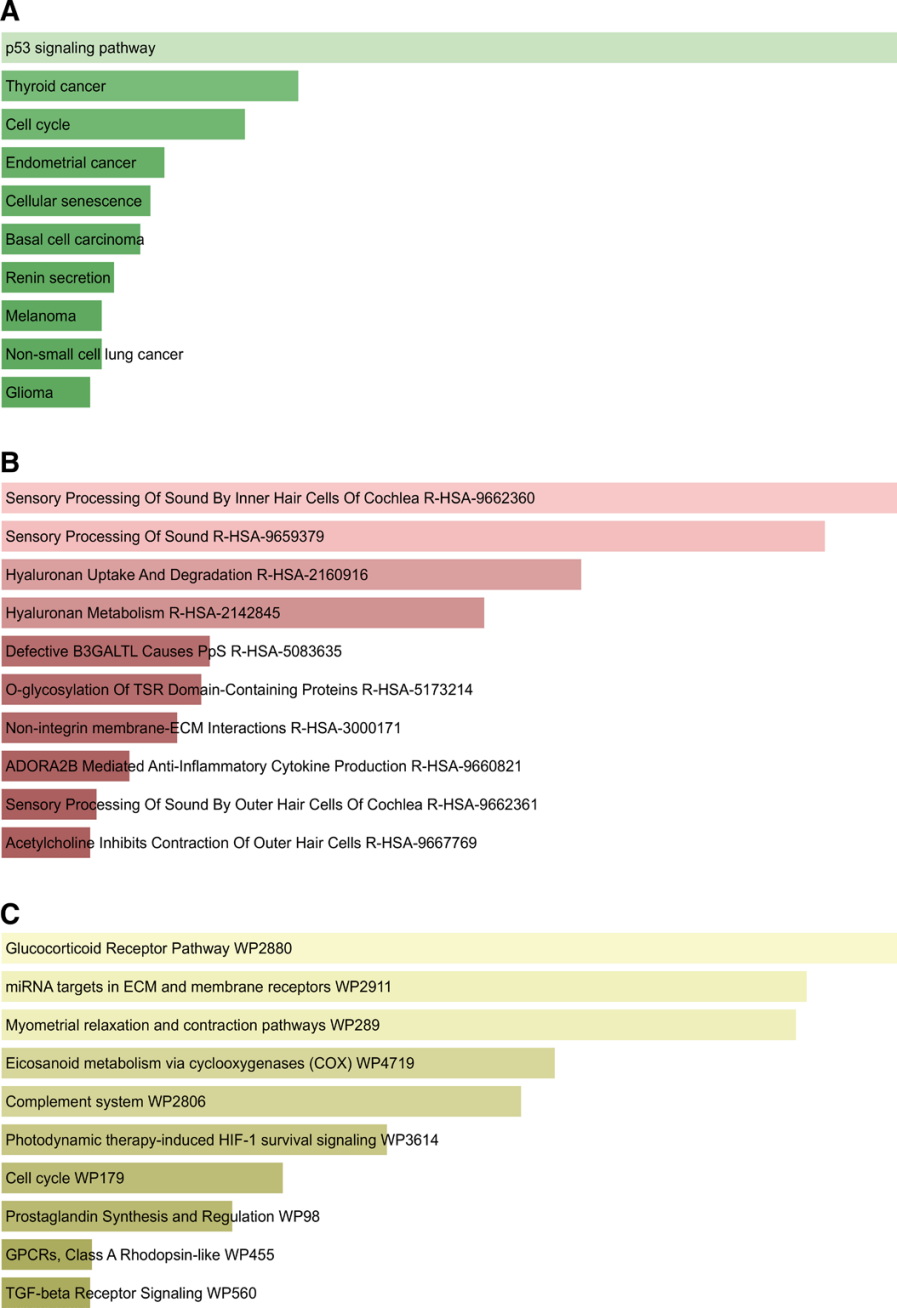
The horizontal bar charts depicting pathway enrichment analysis result of overlapped differentially expressed genes (DEGs) among coronavirus disease 2019 (COVID-19) and metabolic associated fatty liver disease (MAFLD): (A) Kyoto Encyclopedia of Genes and Genomes (KEGG); (B) Reactome; (C) WikiPathway.

Hereafter, we conducted an enrichment analysis utilizing WikiPathways, KEGG, and Reactome databases. The top 10 terms and their particular information of each category are listed in Table 3. Figure 4 shows the consequence of the signaling pathway and biological function analysis. The most significant terms in each category are p53 signaling pathway (KEGG), Sensory Processing of Sound by Inner Hair Cells of Cochlea (Reactome) and Glucocorticoid Receptor Pathway (Wikipathway).

**Table 3:** Top ten terms and their details in pathway enrichment analysis of overlapped differentially expressed genes (DEGs) among coronavirus disease 2019 (COVID-19) and metabolic associated fatty liver disease (MAFLD).

Category	Term	*P* value	Genes
KEGG 2021	p53 signaling pathway	.000209467	GADD45B/SERPINE1/THBS1/GADD45G
	thyroid cancer	.009610983	GADD45B/GADD45G
	cell cycle	.013514334	ESPL1/GADD45B/GADD45G
	endometrial cancer	.022601438	GADD45B/GADD45G
	cellular senescence	.024705564	GADD45B/SERPINE1/GADD45G
	basal cell carcinoma	.026363843	GADD45B/GADD45G
	renin secretion	.031187289	KCNMA1/ADRB2
	melanoma	.03371993	GADD45B/GADD45G
	non-small cell lung cancer	.03371993	GADD45B/GADD45G
	glioma	.036330224	GADD45B/GADD45G
Reactome 2022	sensory processing of sound by inner hair cells of cochlea R-HSA-9662360	1.50E-04	PCLO/KCNMA1/OTOF/XIRP2
	sensory processing of sound R-HSA-9659379	2.33E-04	PCLO/KCNMA1/OTOF/XIRP2
	hyaluronan uptake and degradation R-HSA-2160916	.001016061	STAB2/HMMR
	hyaluronan metabolism R-HSA-2142845	.001828314	STAB2/HMMR
	defective B3GALTL causes PpS R-HSA-5083635	.009610983	ADAMTS17/THBS1
	O-glycosylation of TSR domain-containing proteins R-HSA-5173214	.010118819	ADAMTS17/THBS1
	non-integrin membrane-ECM Interactions R-HSA-3000171	.011712056	NTN4/THBS1
	ADORA2B mediated anti-inflammatory cytokine production R-HSA-9660821	.015642222	ADRB2/GPR84/PTGDR
	sensory processing of sound by outer hair cells of cochlea R-HSA-9662361	.019084362	KCNMA1/XIRP2
	acetylcholine inhibits contraction of outer hair cells R-HSA-9667769	.019842457	KCNMA1
WikiPathway 2021	glucocorticoid receptor pathway WP2880	.002782053	GADD45B/ACKR3/S100P
	miRNA targets in ECM and membrane receptors WP2911	.003465222	COL5A3/THBS1
	myometrial relaxation and contraction pathways WP289	.003557289	RGS16/IGFBP2/GPR182/ACKR3
	eicosanoid metabolism via cyclooxygenases (COX) WP4719	.006391836	PTGDR/PTGES
	complement system WP2806	.006937428	PTX3/VSIG4/THBS1
	photodynamic therapy-induced HIF-1 survival signaling WP3614	.009610983	SERPINE1/IGFBP2
	cell cycle WP179	.012377851	ESPL1/GADD45B/GADD45G
	prostaglandin synthesis and regulation WP98	.013995406	PTGDR/PTGES
	GPCRs, class A rhodopsin-like WP455	.019680793	SUCNR1/ACKR3/ADRB2/PTGDR
	TGF-beta receptor signaling WP560	.019767618	SERPINE1/THBS1

KEGG = Kyoto Encyclopedia of Genes and Genomes, miRNA = micro-RNA.

### 3.3. Demonstration of PPI network and hub genes

**Figure 5.**
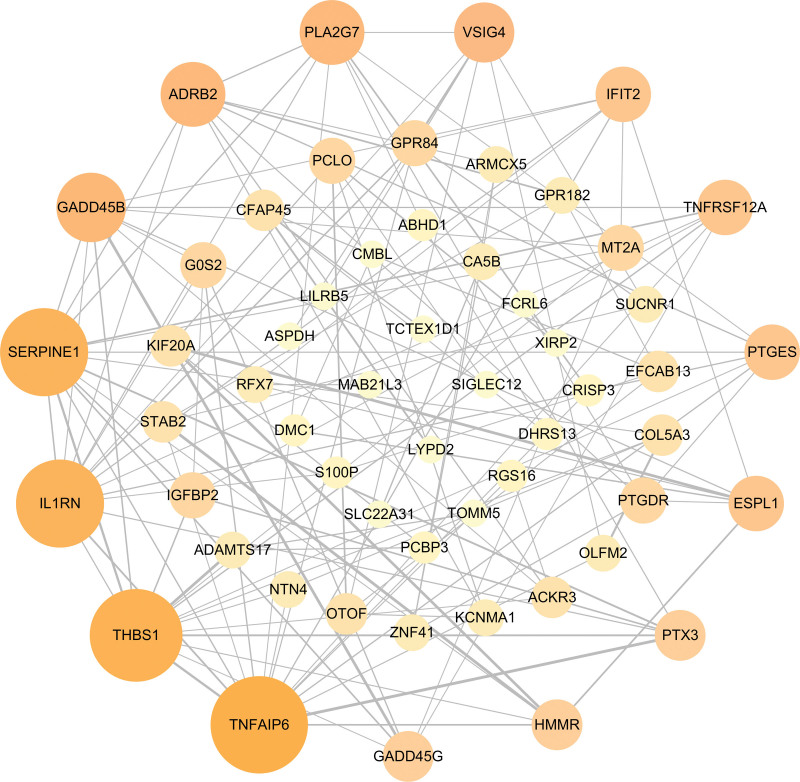
Visualized protein-protein interaction (PPI) network of overlapped DEGs among coronavirus disease 2019 (COVID-19) and metabolic associated fatty liver disease (MAFLD). In the figure, the round nodes symbolize overlapped DEGs, and the edges symbolize the interaction of the nodes. The size of each node represents to the amount of its interactivity. DEGs = differentially expressed genes.

Based on the DEGs data, we used STRING to construct the interaction network of overlapped genes. In the PPI network, the 10 topmost nodes, whose composite scores are larger than 50, were filtrated to present to the hub genes, whose detailed information is listed in Table 4. The 10 core genes include SERPINE1, IL1RN, THBS1, TNFAIP6, GADD45B, TNFRSF12A, PLA2G7, PTGES, PTX3, and GADD45G. To better understand the interaction of hub genes, the visualization of the interaction network (Fig. 5) was conducted, from which we can discover that the hub genes are the most interactive among these genes.

**Table 4:** Hub genes and their detailed information.

Rank	Name	Score
1	SERPINE1	236
2	IL1RN	185
3	THBS1	171
4	TNFAIP6	142
5	GADD45B	84
6	TNFRSF12A	78
7	PLA2G7	77
8	PTGES	74
9	PTX3	54
10	GADD45G	50

### 3.4. Identification of regulation mechanism related to hub genes

**Figure 6.**
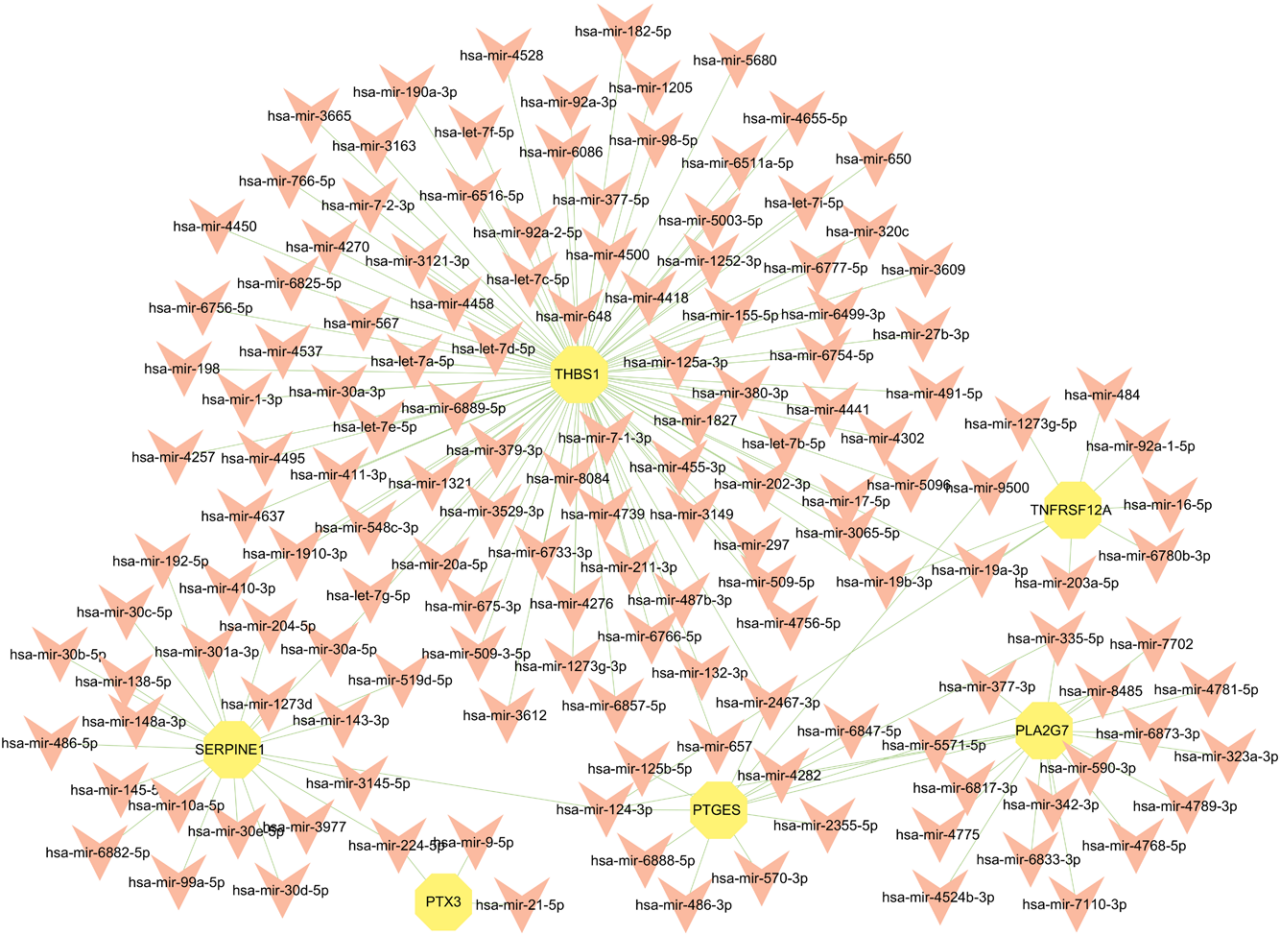
The DEG-miRNA regulatory interaction network. The orange arrow nodes represent to miRNAs, and the yellow circle nodes represent to DEGs. The edges represent to the interaction between miRNA and DEG. miRNA = micro-RNA.

**Figure 7.**
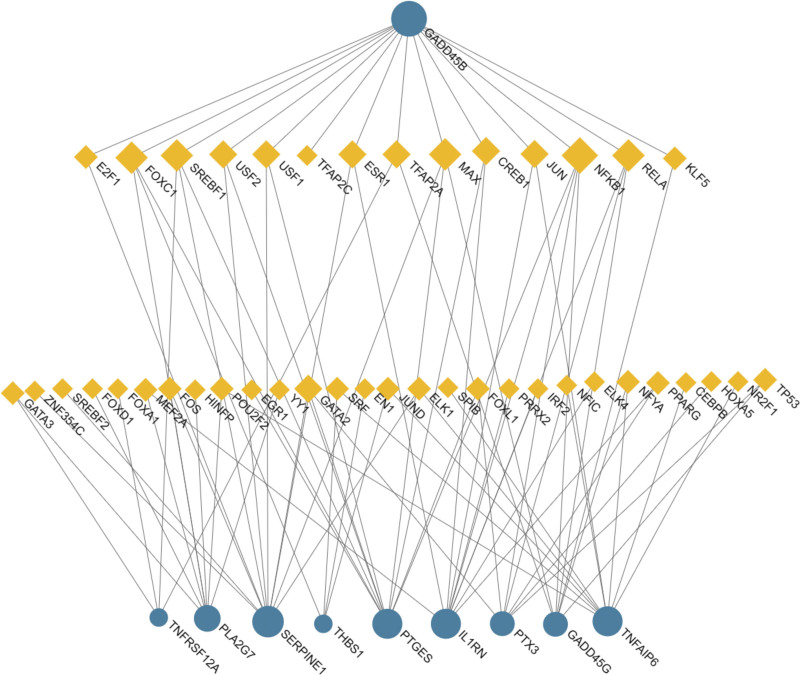
The DEG-TF regulatory interaction network. The yellow square nodes symbolized TFs, and the blue circle nodes symbolized DEGs. The edges represent to the DEG-TF interaction. DEGs = differentially expressed genes, TF = transcription factor.

To better comprehend the regulation process of overlapped DEGs at the transcription level, we utilized the platforms of JASPAR, MiRTarBase, and NetworkAnalyst to construct a regulation network of TFs and miRNA binding to hub genes. Respectively visualized by NetworkAnalyst in Figures 6 and 7, the result of the analysis shows 41 TFs and 151 miRNAs interacting with hub genes.

### 3.5. Assessment of potential drug molecules

Uncovering the drug molecules for the therapy of MAFLD patients infected by SARS-Cov-2 is the most crucial purpose of the study. Utilizing the Enrichr and DSigDB, we filtrated the topmost 10 molecules based on the *P* value of overlapped DEGs. These drugs are 0173570-0000, apigenin, chloropyramine, parthenolide, MS-275, lomustine, raloxifene, terfenadine, luteolin and ciclopirox. Table 5 shows detailed information concerning these drug compounds.

**Table 5:** Detailed information concerning these drug compounds.

Term	*P* value	Genes	Chemical formula
0173570-0000	7.71E-07	SERPINE1/HMMR/ADRB2/KIF20A/THBS1	C_17_H_26_N_2_O_2_
Apigenin	2.50E-06	ARMCX5/SERPINE1/PTX3/ZNF248/ADRB2/THBS1	C_15_H_10_O_5_
Chloropyramine	1.00E-05	GADD45B/SERPINE1/PTX3/ADRB2	C_16_H_20_ClN
Parthenolide	4.72E-05	ESPL1/ARMCX5/RGS16/ACKR3/ZNF248/THBS1	C_15_H_20_O_3_
MS-275	5.53E-05	IL1RN/PCLO/SERPINE1/G0S2/ACKR3/ADRB2/THBS1/PLA2G7/PTGES	C_21_H_20_N_4_O_3_
Lomustine	7.61E-05	ESPL1/G0S2/PTX3/ZNF248/ADRB2	C_9_H_16_ClN_3_O
Raloxifene	8.69E-05	MT2A/RGS16/SERPINE1/G0S2/ACKR3/PTX3/KIF20A/THBS1/PLA2G7/IFIT2/PTGES	C_28_H_27_NOS
Terfenadine	9.76E-05	IL1RN/TNFAIP6/GADD45B/IFIT2	C_32_H_41_NO_2_
Luteolin	1.42E-04	ARMCX5/SERPINE1/ZNF248/ADRB2	C_15_H_10_O
Ciclopirox	7.22E-04	WT2A/TNEAIP6/GADD45B/RGS16/PTX3	C_12_H_17_NO_2_

### 3.6. Identification of disease association

**Figure 8.**
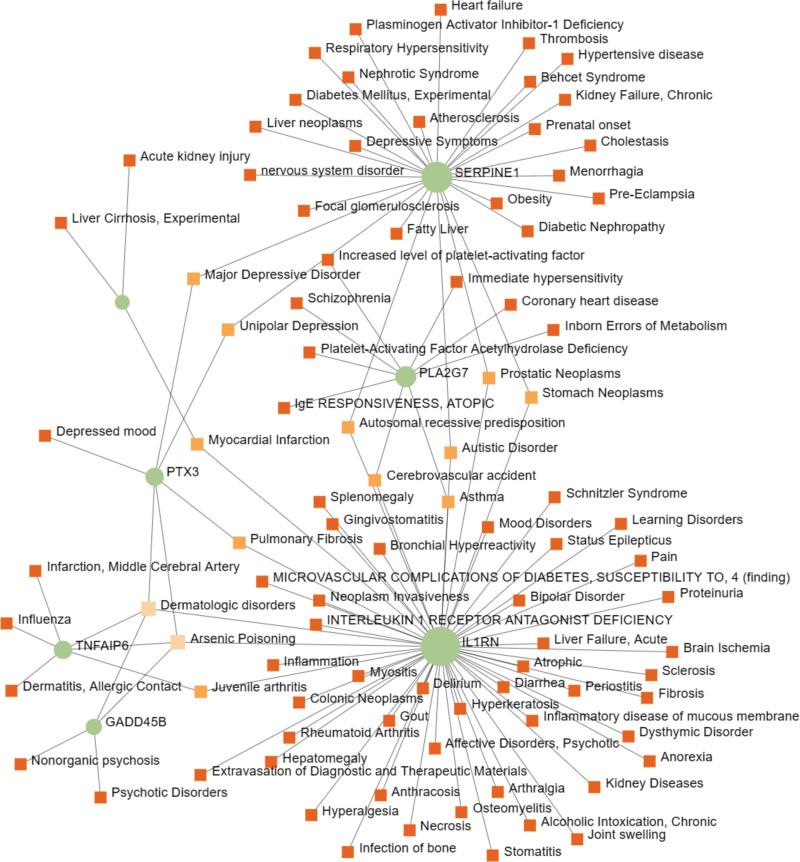
Visualized diseases associated with hub genes among coronavirus disease 2019 (COVID-19) and metabolic associated fatty liver disease (MAFLD) network. In the figure, the green round nodes symbolized hub genes, and the squares nodes symbolized diseases associated with hub genes. The network is produced by DisGeNET and visualized by NetworkAnalyst.

Differentially expressed genes may also be associated with other diseases.^[41]^ Using DisGeNET and NetworkAnalyst, the gene-associated disease analysis, which is visualized in Figure 8, exhibits the network of hub genes among COVID-19 and MAFLD and their related diseases.

## 4. Discussion

The COVID-19 caused by the infection of SARS-CoV-2 can trigger dyspnea, pneumonia, pulmonary fibrosis, and inflammatory cytokine release in different ceases.^[6]^ MAFLD is at risk for further progression to NASH, liver fibrosis, cirrhosis and liver cancer.^[12]^ Inspired by a series of research uncovering the correlation between COVID-19 and MAFLD,^[14–16]^ we conducted an analysis aiming at revealing the influence of COVID-19 on MAFLD by bioinformatics and system biology approach. Expression profiling of high-throughput sequencing datasets is used in biomedical and systems biology research, which has been widely used to identify biomarkers for different diseases.^[42]^ In this study, transcriptome analysis of COVID-19 and MAFLD patients identified 80 shared genes. An in-depth analysis based on shared genes was carried out to further explore the link between MAFLD and COVID-19.

After screening out overlapped DEGs, we performed enrichment analysis to annotate their biological function and signaling pathway. For the part of CC in GO analysis, the most significant terms are tertiary granule lumen and tertiary granule. Granule and granule lumen have an irreplaceable role in the gene translation and matter translocation, and tertiary granules can activate polymorphonuclears (PMNs) by the secreting N-formyl-1-methionyl-1-leucyl-1-phenylalanine and increase PMNs mobility by the gelatinase contained in tertiary granules. Thereafter, PMNs will enter the blood circulation to cope with the infection or inflammation.^[43]^ Given that the most significant symptom in COVID-19 is pneumonia^[44]^ and MAFLD usually accompanies steatohepatitis,^[45]^ we can further put forward a hypothesis that tertiary granules are capable of regulating the immune responses. However, there are only few researches revealing the connection between tertiary granule lumen and COVID-19. Thus, it has the potential to be a new target for therapy.

A PPI network was further constructed based on the common DEGs, with the hub proteins in this network (SERPINE1, IL1RN, THBS1, PLA2G7, PTGES, etc) being regarded as the most critical co-regulator of MAFLD and SARS-CoV-2 infection. Also called PAI1 or PLANH1, SERPINE1 (serpin family E member 1) encodes a member of the serine proteinase inhibitor (serpin) superfamily, which is the suppressor of tissue plasminogen activator (tPA) and urokinase (uPA), and it is also capable of inhibiting the fibrinolysis process.^[46]^ During the epidemic of SARS, Wu et al have already found that the SERPINE1 level raised.^[47]^ A similar elevation reappeared in COVID-19 patients during the pandemic. SERPINE1 levels are generally raised among COVID-19 patients in mild or acute conditions, which indicates that SERPINE1 has a close correlation with the infection of SARS-CoV-2. A study proved this hypothesis. By establishing the lung cell models from human donors, they pointed out that serpin, a cellular protease inhibitor, can act as a blockade of SARS-CoV-2 infection by inhibiting the cellular serine protease, TMPRSS2, to protect target cells from cleavage of the viral spike protein and TMPRSS2-mediated entry.^[48]^ IL1RN, whose full name is interleukin 1 receptor antagonist, is also known as IRAP, IL1F3, IL1RA, etc, and 5 other relevant cytokine genes constitute a gene cluster spanning chromosome. The protein encoded by this gene is a member of the interleukin 1 cytokine family that suppresses the activities of interleukin 1, alpha (IL1A) and interleukin 1, beta (IL1B), while also regulating multiple interleukin-1-related immune and inflammatory responses.^[46]^ Fricke-Galindo et al described that cytokine genes IL1RN could be correlated with disease susceptibility, cytokine storm, and COVID-19 complications.^[49]^ Laterally proving that IL1RN is related to COVID-19 acute case occurrence, the research demonstrated that anakinra, an IL-1 receptor antagonist, improved general survival and invasive ventilation-free survival and was well tolerated in patients with ARDS associated with COVID-19.^[50]^ THBS1 (thrombospondin 1) is also known as TSP1. THBS1 encodes a subunit of a disulfide-linked homotrimeric adhesive glycoprotein that mediates cell-to-cell interactions and cell-to-matrix. This adhesive glycoprotein can influence platelet aggregation, angiogenesis, and tumorigenesis.^[51]^ An in vivo experiment indicated that the clearance of THBS1 can mitigate the early stages of fibrogenesis,^[52]^ possibly relating to the progress of MAFLD. PLA2G7 (phospholipase A2 group VII) is also called PAFAD, or LP-PLA2. It encodes a secreted enzyme whose function is to catalyze the degradation of platelet-activating factors to biologically inactive products.^[53]^ PLA2G7 was found to be mainly expressed by proinflammatory macrophages in lungs appeared after the infection of SARS-CoV-2. Moreover, serum protein levels of PLA2G7 were extremely high and exceeded the standard range in COVID-19 patients, particularly among the patients.^[54]^ Therefore, PLA2G7 is very a promising prognostic and therapeutic target for the therapy of COVID-19. PTGES (prostaglandin E synthase) is also named prostaglandin E synthase type 2 and MPGES. A series of published proteomic research on the interaction among SARS-CoV-2 and human host proteins indicated that the inhibition of prostaglandin E synthase type 2, which PTGES2 encodes, can suppress SARS-CoV-2 activity.^[55–57]^ Thus, PTGES is supported to be a potential target for antiviral drugs.

In the context of miRNA, we screened out 151 miRNAs interacting with hub genes most, which consisted of hsa-mir-124-3p, hsa-mir-30a-5p, hsa-mir-2467-3p, hsa-mir-19a-3p, hsa-mir-19b-3p, hsa-mir-224-5p, hsa-mir-335-5p, the miRNAs. A literature pointed out that the regulation of has-mir-124-3p can improve abnormal lipid metabolism in the liver.^[58]^ The content of hsa-mir-30a-5p, hsa-mir-19a-3p hsa-mir-19b-3p in serum grows higher in COVID-19 patients,^[59,60]^ while the content of miR-224-5p, a targeting pro-inflammatory factors, decreased.^[61]^ The expression levels of hsa-mir-19a-3p up-regulated and hsa-mir-335-5p are up-regulated in MAFLD patients.^[62]^

Determining potential drug compounds is the most critical part of this research. Parthenolide is a germacrane sesquiterpene lactone and can be extracted from the traditional medicine plant Tanacetum parthenium and displays anti-inflammatory activity.^[63]^ By the fact that hepatic inflammation drives the progression of MAFLD toward cirrhosis^[64]^ and COVID-19 may contribute to systemic inflammation that may lead to acute respiratory distress, multiple organ failure and even death in some patients,^[65]^ parthenolide is worth to be taken into consideration to take in practice. Luteolin is a tetrahydroxy flavone that can act as an antioxidant, an anti-inflammatory agent, and an immune system modulator.^[66]^ A study found that Long-COVID syndrome symptoms, especially brain fog, can be mitigated by taking luteolin,^[67]^ and Qi-Dong Xia et al found that Lianhua Qingwen capsule, a Chinese patent drug, has shown therapeutic effects in patients with COVID-19, while luteolin is one of its main active compounds. Moreover, luteolin can mitigate NASH, a stage of MAFLD, by targeting the pro-inflammatory IL-1 and IL-18 pathways.^[68]^ All these facts indicate that luteolin could be a target drug compound for MAFLD and COVID-19 comorbidity patients. Showing significant SARS-CoV-2 3-chymotrypsin like protease (3CLpro) inhibiting activity, apigenin is a kind of flavonoid purified from ethanolic leaf extracts of Torreya nucifera, a common traditional Chinese medicine.^[69]^ 3CLpro is one of the most crucial proteases produced by SARS-CoV-2. It takes part in the proteolysis of nonstructural proteins which will, ultimately, form 16 nonstructural proteins, including Helicase and RNA-dependent RNA polymerase, that participate in the transcription and duplication. Therefore, with the capability of inhibiting the 3CLpro, apigenin has a remarkable potential to be an anti-SARS-CoV-2drug candidate.^[70]^ Furthermore, a study demonstrated that apigenin could decrease hepatic lipid accumulation by activating the autophagy-mitochondrial pathway.^[71]^ The studies abovementioned convincingly suggested the probability of the usage of apigenin in SARS-CoV-2-infected MAFLD. MS-275, also called Entinostat, is a synthetic benzamide derivative. A study proposed that MS-275 is able to down-regulate the expression of ACE-2,^[72]^ which means it can target the process of SARS-CoV-2 entry. Qi Zhang et al found that MS-275 can induce the hepatic fibroblast growth factor 21 (FGF21) expression.^[73]^ FGF21 can regulate lipid metabolism and improves insulin resistance, which is one of the causes of MAFLD.^[74]^ In a phase 2 study, hepatic steatosis is decreased after 16 weeks administering pegbelfermin, a FGF21 analogue, to patients with NASH.^[74]^ Thus, MS-275 has the huge potential to cure MAFLD and COVID-19 comorbidity patients.

Ultimately, we conducted an analysis to screen out diseases related to hub genes. The result depicts that mental disorder, including autistic disorder, depressive symptoms, schizophrenia, etc, is the most significant disease type. Literature indicated that COVID-19 and psychiatric disorders could worsen each other.^[75,76]^ The hub gene also mainly correlated to inflammation symptoms consisted of immediate hypersensitivity myositis, osteomyelitis, stomatitis, etc. Research proved that both MAFLD and COVID-19 could lead to systematic inflammation.^[77–80]^ Liver-associated diseases, like liver neoplasms, acute liver failure, fatty liver, and liver cirrhosis, also took a significant share in the analysis result. This association between liver disease and hub genes may account for the existence of the mutual promotion between COVID-19 and MAFLD.^[14–17]^

In this study, we filtrated shared DEGs between MAFLD and COVID-19, annotated them with the biological function and signaling pathways, identified hub genes, and screened out the potential drug molecules. However, our study also has some limitations. Bioinformatics is not able to replace the clinical test, and further basic experiments are necessary to verify our findings. Besides, the further clinical trials are also needed for verifying the effectiveness and safety of drug candidates, which will be the focus of our following tasks.

## 5. Conclusion

We performed an analysis with COVID-19 datasets and MAFLD datasets from GEO. Bioinformatic and system biology analysis identified the DEGs in each dataset and filtrated 80 overlapped DEGs. Then we annotated those overlapped DEGs with the biological function and signaling pathways and constructed a PPI network. Based on PPI network, we extracted the topmost 10 hub genes with the highest interactivity and found 109 diseases related to hub genes. Last, we screened out 10 potential drug molecules for the therapy of COVID-19 and MAFLD comorbidity patients. The research provides a potential direction for further investigation of the molecular mechanisms and treatment of COVID-19 and MAFLD.

## Acknowledgments

The authors acknowledge all patients who donate samples, the contributors who upload datasets and the GEO database for providing platforms.

## Author contributions

**Formal analysis:** Yujia Song.

**Investigation:** Yuchu Xiang.

**Visualization:** Yujia Song.

**Writing – review & editing:** Hongyuan Pan, Fang Wang.

**Writing – original draft:** Tengda Huang, Dawei Zheng, Guoteng Qiu, Zichen Wang.
